# Dual-responsive nanosystem based on TGF-β blockade and immunogenic chemotherapy for effective chemoimmunotherapy

**DOI:** 10.1080/10717544.2022.2069877

**Published:** 2022-05-04

**Authors:** Xiaoxian Huang, Lingfei Han, Ruyi Wang, Wanfang Zhu, Ning Zhang, Wei Qu, Wenyuan Liu, Fulei Liu, Feng Feng, Jingwei Xue

**Affiliations:** a Department of Natural Medicinal Chemistry, China Pharmaceutical University, Nanjing, China; b Department of Pharmaceutical Analysis, China Pharmaceutical University, Nanjing, China; c Zhejiang Center for Safety Study of Drug Substances (Industrial Technology Innovation Platform), Hangzhou, China; d Tumor Precise Intervention and Translational Medicine Laboratory, Taian City Central Hospital, Taian, China; e Pharmaceutical Department, Taian City Central Hospital, Taian, China; f Jiangsu Food and Pharmaceutical Science College, Huaian, China

**Keywords:** Chemoimmunotherapy, TGF-β blockade, immunosuppression tumor microenvironment, immunogenic cell death, nanosystem

## Abstract

The antitumor immune response induced by chemotherapy has attracted considerable attention. However, the immunosuppressive tumor microenvironment hinders the immune activation effect of cancer chemotherapy. TGF-β plays a key role in driving tumor immunosuppression and can prevent effective antitumor immune response through multiple roles. In this study, a dual-responsive prodrug micelle (PAOL) is designed to co-deliver LY2109761 (a TGF-β receptor I/II inhibitor) and oxaliplatin (OXA, a conventional chemotherapy) to remodel tumor microenvironment and trigger immunogenic cell death (ICD) to induce antitumor immunity response. Under hypoxia tumor environment, the polyethylene glycol shell of the micelle cleavages, along with the release of LY2109761 and OXA prodrug. Cytotoxic effect of OXA is then activated by glutathione-mediated reduction in tumor cells and the activated OXA significantly enhances tumor immunogenicity and promotes intratumoral accumulation of cytotoxic T lymphocytes. Meanwhile, TGF-β blockade through LY2109761 reprograms tumor microenvironment by correcting the immunosuppressive state and regulating tumor extracellular matrix, which further maintaining OXA induced immune response. Therefore, due to the capability of boosting tumor-specific antitumor immunity, the bifunctional micelle presents markedly synergistic antitumor efficacies and provides a potent therapeutic strategy for chemoimmunotherapy of solid tumors.

## 1. Introduction

Chemotherapy, which can directly inhibit the proliferation or cause the death of malignant cells, is one of the most important treatments for cancers in clinic (Chen et al., 2019; Galluzzi et al., 2020). Emerging evidence suggests that certain conventional chemotherapeutics, such as oxaliplatin (OXA) and doxorubicin, can induce an additional tumor controlling effect called immunogenic cell death (ICD) (Wang et al., 2018), which is a particular manner of cell death and are mainly characterized by damage-associated molecular patterns (DAMPs) (Krysko et al., 2012). However, due to the immune escape hallmark of cancer (Hanahan & Weinberg, 2011), this triggered antitumor immune response is always hindered by immunosuppressive tumor microenvironment (Qiao et al., 2018; Dai et al., 2021).

Tumor microenvironment is a complex ecosystem containing various cellular elements (tumor cells, immune cells, fibroblasts, and stromal cells) (Hui & Chen, 2015) as well as extracellular components (cytokines, extracellular matrix (ECM), etc.) (Wu & Dai, 2017; Hu et al., 2021). It always tends to be immunosuppressive state due to the upregulated immunosuppressive cytokines, especially for transforming growth factor-β (TGF-β) (Qiao et al., 2018). TGF-β is a pleiotropic cytokine which can not only foster tumor progression and metastasis, but also play key role in facilitating the formation of immunosuppressive tumor microenvironment (Mariathasan et al., 2018). Many tumors including cancers of breast, colon, and pancreas overexpress TGF-β (Wrzesinski et al. 2007). It prevents effective antitumor immune response through multiple roles, including inhibiting the generation and function of effector T cells, natural killer (NK) cells, and antigen-presenting dendritic cells (DCs) (Flavell et al., 2010; Batlle & Massague, 2019), dampening the inflammatory functions of tumor-associated macrophages (TAMs) (Chiara Porta et al., 2009), promoting the expansion of T regulatory cells (Tregs) (Flavell et al. 2010). Moreover, TGF-β signaling can induce the production of tumor stroma content, expression of ECM proteins and fibroblast activation (Lan et al., 2018), which further impede antitumor immunity by forming a physical barrier for the infiltration of immune cells (Huang et al., 2020). Furthermore, inhibition of TGF-β signaling has been evaluated in multiple clinical trials to improve the therapeutic effect of cancer immunotherapies (Batlle & Massague, 2019; Derynck et al., 2021). Therefore, due to the central role of TGF-β in the immunosuppressive tumor microenvironment (Tauriello et al., 2022), blockade of TGF-β signaling pathway to remodel the tumor microenvironment would be a promising strategy to enhance chemoimmunotherapy efficiency.

Hypoxia is one of the main pathological features of most solid tumor microenvironment (Wilson & Hay, 2011; Yang et al., 2019) and the hypoxia tumor cells lead to immunosuppression tumor microenvironment by accumulating immunosuppressive cells and secreting immunosuppressive factors, such as TGF-β (Yang et al., 2021). Besides, the ECM around tumor cells contributes to tumor hypoxia and retards the interstitial delivery of nanodrugs (Qiao et al., 2022). More importantly, TGF-β is the major cytokine that upregulates tumor ECM and inhibiting TGF-β can reduce collagen deposition (Qiao et al., 2022). Hence, regulating TGF-β at the hypoxia tumor microenvironment would be an efficient strategy for remodeling tumor environment to improve chemoimmunotherapy.

**Figure 1.**
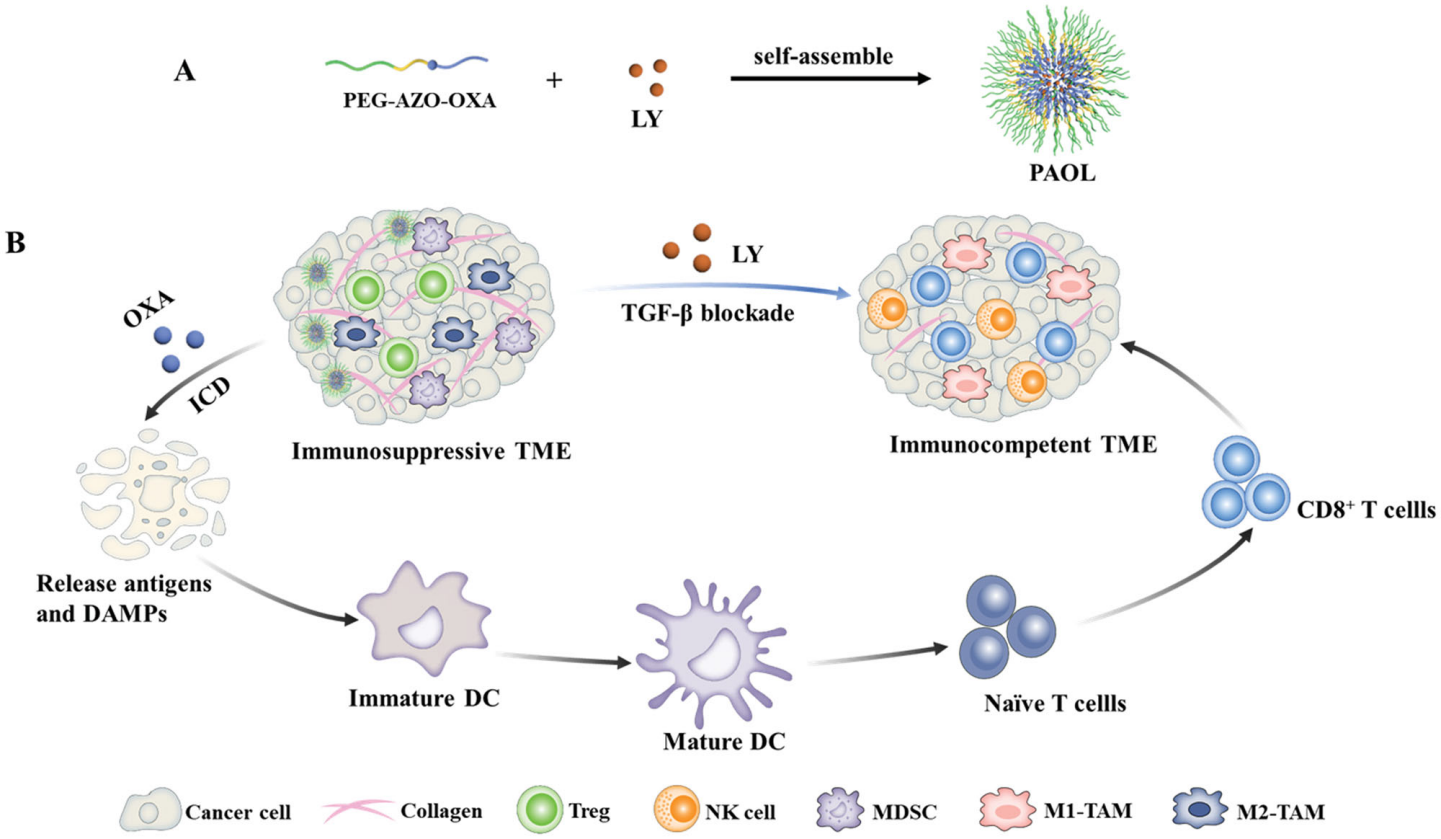
The scheme illustrating the synergistic therapeutic efficacy of the bifunctional PAOL. (A) The self-assembly procedure of PAOL nanosystem. (B) LY could be released to remodel the tumor microenvironment by the blockade of TGF-β signaling. The released oxaliplatin could induce ICD effect and promote DC maturation to prime robust antitumor immune response. Combination with oxaliplatin and TGF-β blockade strategy synergistically could effectively prevent the tumor growth and metastasis.

The antineoplastic chemotherapeutic OXA has been proven the capacity of inducing antitumor immune response by ICD (Krysko et al. 2012; Stojanovska et al., 2019). LY2109761 (LY) is a TGF-β receptor I/II kinase inhibitor and exhibits antitumor activity in various cancers (Lahn et al., 2015). Combination of LY with chemotherapy has been reported in the ability of impeding tumor progression and metastasis (Li et al., 2019). Herein, as shown in Figure 1, we developed a tumor hypoxia and reduction microenvironment dual-responsive nano micelles to co-deliver LY and OXA to remodel tumor microenvironment and induce antitumor immune response. A hypoxic sensitive linker, azobenzene, was used to connect OXA prodrug with a polyethylene glycol (PEG) coating. LY was encapsulated into the hydrophobic core of the micelles. Upon reaching the tumor site through the enhanced permeation and retention (EPR) effect, the nitrogen–nitrogen double bond would break under the hypoxia microenvironment. The micelles disassembled and LY released, together with releasing OXA prodrug and the PEG corna. LY could effectively block the TGF-β signaling to relive immune suppression and regulate tumor ECM. The OXA prodrug could be activated in the reduction microenvironment of tumor cells to trigger ICD effect and induce antitumor immunity. As a result, the dual-responsive nanosystem represents a significant tumor inhibition effect through remodeling the tumor microenvironment and inducing ICD to improve chemoimmunotherapy.

## 2. Materials and methods

### 2.1. Materials

OXA was purchased from Platinum Energy Co. Ltd. (Shandong, China). Ethyl-3-(3-dimethyl amino propyl) carbodiimide hydrochloride (EDCI), Methoxypolyethylene glycol amine (PEG_2k_-NH_2_), N-Boc-ethylenediamine, and 4-dimethylaminopyridine (DMAP) were bought from the Aladdin Industrial Corporation (Shanghai, China). Stearic anhydride, trifluoroacetic acid (TFA), glutathione (GSH), and N,N-dimethylformamide (DMF) were purchased from the Energy Chemical Corporation (Shanghai, China). Succinic anhydride was purchased from Sinopharm Chemical Reagent. 4′,6-Diamidino-2-phenylindole (DAPI), 3-(4,5-dimethyl-2-thiazolyl)-2,5-diphenyl-2-H-tetrazolium bromide (MTT), and RPMI 1640 cell culture medium were purchased from KeyGen Biotech. (Nanjing, China). 1,1′-Dioctadecyl-3,3,3′,3′-tetramethylindotricarbocyanineiodide (DiR) was obtained from Life Technologies (Shanghai, China). LY2109761 (LY) was purchased from TopScience (Shanghai, China). The ATP Assay Kit was obtained from Beyotime (Shanghai, China). Fetal bovine serum (FBS) was purchased from Biological Industries (BI, Cromwell, CT). Collagenase type IV was purchased from Gibco (Carlsbad, CA). Anti-CD11c-APC, anti-CD80-BV421, and anti-CD86-PE were purchased from BD Biosciences (Franklin Lakes, NJ). Anti-CD25-BV650 was obtained from Biolegend (San Diego, CA) and other antibodies for flow cytometry were purchased from eBioscience (San Diego, CA). All other reagents and solvents mentioned in this article were of analytical grade.

### 2.2. Cell culture and mouse model

4T1 breast cancer cells and CT26 colorectal cancer cells were obtained from the cell bank of the Chinese Academy of Sciences (Shanghai, China). Both cell lines were cultured in complete RPMI 1640 cell culture medium containing 10% FBS and maintained at 37 °C in 5.0% CO_2_ atmosphere. Hypoxic culture conditions (1% O_2_, 5% CO_2_, 37 °C) were mimicked using a hypoxic cell culture chamber system (MAWORDE, Beijing, China).

Six to eight weeks old female Balb/c mice were purchased from the Institute of Comparative Medicine Yangzhou University, Yangzhou, China. All animal experiments were performed according to the guidelines for laboratory animals established by China Pharmaceutical University, Nanjing, China. 1 × 10^6^ 4T1 or CT26 cells were injected into the left flank of the mice to establish the tumor-bearing model. Tumor volume was measured every other day and calculated by the following formula: (length × width^2^)/2.

### 2.3. Synthesis, preparation, and characterization of PAOL

The synthetic procedures for PEG-AZO-OXA are detailed introduced in the Supporting Information and the PAOL micelles were prepared by the solvent evaporation method (Li et al. 2019). Briefly, the PAOL was prepared as follows: PEG-AZO-OXA (10 mg) and LY (4.5 mg) were dissolved in dichloromethane and condensed by vacuum evaporation. Then, 1 mL water was added and sonicated to form the LY loaded PAOL micelles. PAO was prepared via the aforementioned procedure without addition LY. The drug loading (DL%) of LY was detected by high-performance liquid chromatography (HPLC) and the Pt concentration was recorded by inductively coupled plasma-mass spectrometry (ICP-MS).

The particle size distribution and surface charge of PAOL were determined using dynamic light scattering (DLS) by a Malvern Zetasizer Nano ZS (Worcestershire, UK). Furthermore, the morphology of PAOL was observed by transmission electron microscopy (TEM, HT7700, Hitachi, Chiyoda, Japan). To evaluate the physiological stabilities of PAOL, the hydrated diameters were recorded after being incubated in PBS and 10% FBS containing solution for time intervals.

The critical micelle concentration (CMC) of PAO was measured by the classic fluorescence pyrene method according to our previous study (Han et al., 2019).

The drug release profiles *in vitro* were investigated at different buffer solutions. Sodium dithionite (10 mM) and GSH (10 mM) were used to mimic the hypoxic (Guo et al., 2020) and reducing condition. Two milliliters solution of PAOL was dialyzed in the normoxia condition with or without GSH and hypoxia condition with or without GSH at 37 °C under shaking. At the predetermined time intervals, the released amounts of Pt and LY were quantified by ICP-MS and HPLC as aforementioned.

### 2.4. Cytotoxicity and cellular uptake *in vitro*

The cytotoxicity was measured using the methyl thiazolyl tetrazolium (MTT) assay. 4T1 cells were seeded in the 96-well plate at a density of 5 × 10^3^ cells per well for 12 h, the cells were incubated with free OXA and PAOL at different concentrations of Pt under standard culture condition or hypoxia condition for 48 h before MTT assay.

To evaluate the cellular uptake of PAOL, 4T1 cells were seeded in the six-well plate at the density of 1 × 10^5^ cells per well for 24 h. Subsequently, cells were incubated with free OXA under hypoxia culture condition and PAOL (Pt = 10 μM) under hypoxia or standard condition for 24 h. Then, the cells were washed with PBS for three times and collected. Then, the uptake of Pt into cells was measured by ICP-MS and the protein was quantified by the bicinchoninic acid (BCA) method.

### 2.5. Immunogenic cell death induction of PAOL *in vitro*

The ATP secretion was tested using a commercially available ATP assay kit. Briefly, 4T1 cells were seeded in the 24-well plate at a density of 5 × 10^4^ cells per well for 12 h and treated with free OXA and PAOL (Pt = 10 μM) under hypoxia culture condition and PAOL in normoxia culture condition. After 24 h, the cell culture supernatant was collected and the ATP contents were quantified according to the manufacturer’s instructions of the kit.

Immunofluorescence analysis was used to detect the calreticulin (CRT) and intracellular HMGB1 distribution. For the surface detection of CRT, 4T1 cells were seeded on a glass bottom dish at a density of 2 × 10^4^ cells per well for 12 h and then treated with free OXA and PAOL under hypoxia culture condition and PAOL (Pt = 10 μM) in normoxia culture condition for 24 h. Next, the cells were washed with PBS and fixed with 4% paraformaldehyde before incubated with anti-CRT primary antibody and Alexa488-conjugated monoclonal secondary antibody. Finally, the cells were stained with DAPI and examined by the confocal laser scanning microscope (CLSM, Zeiss, LSM 800, Oberkochen, Germany).

To detect the intracellular HMGB1, 4T1 cells were incubated with free OXA and PAOL under hypoxia culture condition and PAOL (Pt = 10 μM) in normoxia culture condition for 24 h. Next, the cells were washed with PBS, fixed with 4% paraformaldehyde and permeabilized with 0.1% Triton X-100. Nonspecific binding sites were blocked with 5% FBS before incubated with anti-HMGB1 primary antibody and Alexa488-conjugated monoclonal secondary antibody. Finally, the cells were stained with DAPI and observed by the CLSM.

### 2.6. DC maturation *in vitro*

Bone marrow-derived dendritic cells (BMDCs) were isolated and generated from the bone marrow of 6–8 weeks old Balb/c mice. Bone marrow was collected and cultured with RPMI 1640 medium with 10% FBS, 20 ng/mL murine GM-CSF (PeproTech, Cranbury, NJ) and 20 ng/mL IL-4 (PeproTech, Cranbury, NJ). New medium was added on the day 3 and the non-adherent and loosely adherent cells were harvested after another four days. 4T1 cells were pretreated with free OXA and PAOL under hypoxia culture condition and PAOL (Pt = 10 μM) in normoxia culture condition for 24 h. Subsequently, 1 × 10^6^ immature DC cells were co-cultured with the pretreated 4T1 cells for 24 h. Then, the DC cells were stained with anti-CD11c-APC, anti-CD80-BV421, and anti-CD86-PE antibodies, and the maturation of DC cells were evaluated by using flow cytometry measurement (FCM) (BD LSRFortessa, Franklin Lakes, NJ).

### 2.7. Biodistribution *in vivo*

4T1 tumor-bearing models were used to investigate the biodistribution of DiR@PAO. When the tumor volume reached about 200 mm^3^, the tumor-bearing mice were grouped randomly and were intravenously injected via the tail vein with free DiR and DiR@PAO and then observed at predetermined time intervals postinjection with IVIS spectrum small-animal imaging system (PerkinElmer, Waltham, MA). The mice were sacrificed after injected for 24 h and the main organs including heart, liver, spleen, lung, kidney, and tumors were collected and observed *ex vivo* to analyze the biodistribution profile.

### 2.8. Antitumor activity and antimetastasis effect *in vivo*

The antitumor activity *in vivo* was evaluated in both 4T1 tumor-bearing mice and CT26 tumor-bearing mice. 4T1 or CT26 tumor-bearing mice were randomly divided into six groups when the tumor volume reached about 100 mm^3^. The mice were treated with saline, LY, OXA, OXA + LY, PAO, and PAOL at an equal Pt dose of 2.5 mg/kg and LY dose of 8 mg/kg. All the administration were given at a time interval of two days for five times. The weight of the mice and the length and width of the tumors were recorded every other day for 21 days. The mice were sacrificed on day 21, the tumor and the lung were harvested, fixed in 4% paraformaldehyde solution and the tumors were weighed. H&E staining and terminal deoxynucleotidyl transferase mediated nick end labeling (TUNEL) assay were used to assess histological analysis and apoptosis levels in tumors. Immunohistochemistry assay of α-smooth muscle actin (α-SMA) and p-Smad2, as well as Picrosirius Red staining were used to analyze the 4T1 tumor tissues.

### 2.9. Antitumor immune response analysis *in vivo*

The 4T1 tumors were harvested at two days post the treatment and the histological sections were performed by immunofluorescence staining. The tumor sections were stained with anti-CRT and anti-HMGB1 antibodies and then treated with the secondary antibodies to analyze the CRT and HMGB1 expression.

The tumor-draining lymph nodes and tumors were harvested for immune analysis at two days post the treatments. The lymph nodes were homogenized to prepare single-cell suspension. The cell suspensions were stained with live/dead, anti-CD11c-APC, anti-CD80-BV421, and anti-CD86-PE antibodies and the analysis of mature DCs (CD80^+^CD86^+^ in CD11c^+^ DCs) was evaluated by FCM. The tumors were cut into small pieces, digested with 1 mg/mL collagenase IV and collected by centrifugation. The single-cell suspensions were first stained with live/dead. Then anti-CD3-APC-eFluor 780, anti-CD4-Percp-Cy5.5, anti-CD8-FITC, and anti-CD25-Brilliant Violet 650 antibodies were incubated with the suspensions to analysis CTLs (CD3^+^CD8^+^) and Tregs (CD3^+^CD4^+^CD25^+^).

To analyze tumor-associated myeloid-derived suppressor cells (MDSCs), the tumor single-cell suspensions were also stained with anti-CD11b-APC-Cy7 and anti-Gr1-Brilliant Violet 650. Besides, NK cells (CD3^–^NK1.1^+^) and TAMs (CD11b^+^F4/80^+^) were also been tested. Moreover, interferon gamma (IFN-γ) and tumor necrosis factor α (TNF-α) in the serum were determined using the enzyme-linked immunosorbent assay (ELISA) assay under the kit instructions.

### 2.10. Safety evaluation *in vivo*

Balb/c female mice were divided into six groups randomly and various administrations were treated for five times at intervals of two days. Mice were sacrificed on day 11 after treatment. The blood was collected and plasma was used to perform blood chemistry analysis and the major organs (heart, liver, spleen, lung, and kidney) were collected for H&E staining to monitor the morphological and pathological feathers.

### 2.11. Statistical analysis

Data are presented as mean ± standard deviation. Statistical significance was tested using a two-tailed Student's *t*-test. The significance threshold was *p< .05, **p< .01, and ***p< .001.

## 3. Results and discussion

### 3.1. Synthesis and characterization of PAOL nanosystem

In this study, the synthetic procedures for PEG-AZO-OXA are shown in the Supporting Information Figures S1–3. OXA (IV) precursors 1–2 were prepared according to the previously reported routes (Feng et al., 2016; Chen et al., 2019). Afterwards, the long hydrophobic chain and the linker with a terminal amino group were reacted with the OXA (IV) precursor respectively to obtain the hydrophobic part. The chemical structure of every intermediate product was confirmed by ^1^H NMR (Figures S4–6,8,10) and mass spectroscopy (Figures S7,9,11). Amine-terminated PEG was reacted with azobenzene-4,4′-dicarboxylic acid to provide the hydrophilic and hypoxia-responsive part. The terminal prodrug PEG-AZO-OXA was employed the condensation reaction of amino and carboxyl groups. The chemical structure of PEG-AZO-COOH and PEG-AZO-OXA was confirmed by ^1^H NMR (Figures S12, 13).

**Figure 2.**
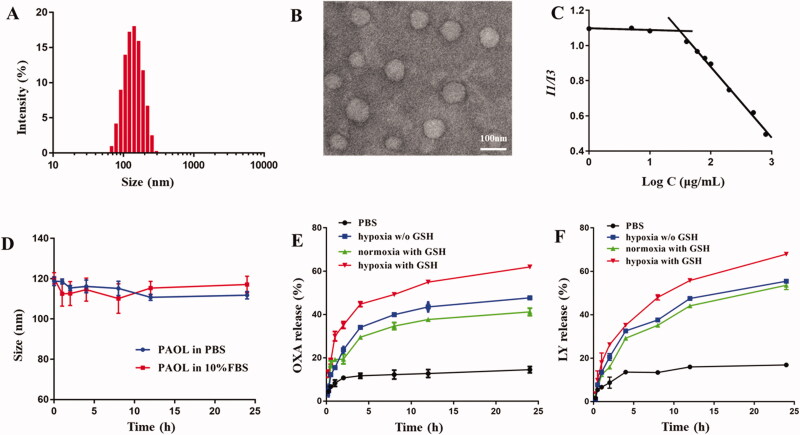
Characterization of PAOL. (A) Size distribution and (B) TEM image of PAOL. (C) CMC value of PEG-AZO-OXA. (D) Size changes of PAOL in PBS (pH 7.4) and 10% FBS over 24 h. (E) OXA and (F) LY release profiles of PAOL at mimicked hypoxia (10 mM sodium dithionite) and normoxia condition, in the presence or absence of 10 mM GSH (w/o GSH means without GSH). Data are presented as mean ± SD (*n* = 3).

Due to the amphiphilic characteristic of PEG-AZO-OXA, it could self-assemble into PAO micelles. Hydrophobic cargo, LY was loaded in the PAOL micelles. The DL% of Pt in the polymer was approximately 6.31% and the DL% of LY was 16.8%. The morphology, particle size, ζ-potential, and CMC were all evaluated to study the characteristics of the micelles. The hydrodynamic size of PAOL was 114.6 ± 1.6 nm measured by DLS (Figure 2(A)) and the well-dispersed spherical morphology was confirmed by TEM (Figure 2(B)). A nearly neutral surface charge was shown due to the methoxyl PEG. Besides, the CMC was determined by fluorescence at 30.97 μg/mL (Figure 2(C)), which could maintain good stability in blood circulation. In addition, the PAOL showed good stability in both pH 7.4 PBS and 10% FBS media (Figure 2(D)), which is beneficial for drug delivery *in vivo*.

Hypoxia is a main pathological feature of most solid tumor microenvironment and the concentration of GSH within tumor cells is much higher than the extracellular environment (Yang et al., 2021). To confirm the hypoxia-responsiveness and reduction-activated of drug release, sodium dithionite (10 mM) and GSH (10 mM) were used to mimic the hypoxic (Guo et al., 2020) and reducing condition. Sodium dithionite could reduce the nitrogen–nitrogen double bond accurately and the link of azobenzene between PEG and OXA prodrug was cleaved. As shown in Figure 2(E,F), the release of both Pt and LY in pH 7.4 PBS displayed a minimal release. In contrast, PAOL performed a significant sustained release property under hypoxia and reduction condition, indicating that the micelles disassembly and the cleavage of the PEG shell. The reason why Pt could also release under hypoxia condition without the addition of GSH might be that the micelles disassembled and the released OXA (IV) prodrug could also be dialyzed and measured. The drug release profile indicated that the cleavage of the PEG shell under hypoxia condition and LY released together with the OXA (IV) prodrug. The OXA (IV) prodrug release of OXA is dependent upon the reduction condition.

### 3.2. Cellular uptake and cytotoxicity

**Figure 3.**
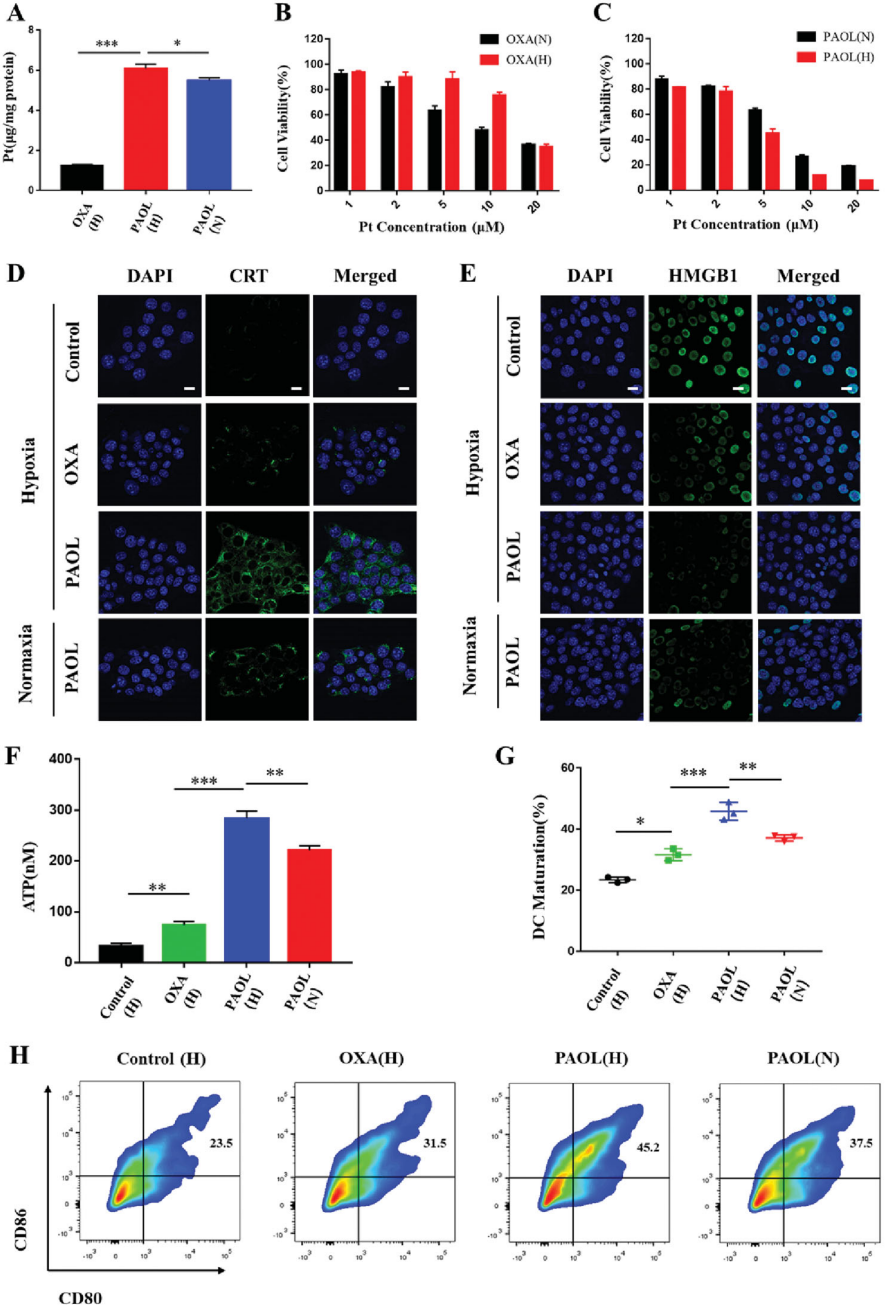
*In vitro* cellular experiments. (A) Cellular uptake of free OXA under hypoxia and PAOL under normoxia and hypoxia. (H) Represents cells incubated under hypoxia and (N) represents cells under normoxia. Cell viability of 4T1 cells examined post 48 h incubation with (B) OXA and (C) PAOL under hypoxia and normoxia condition. DAMPs of (D) CRT, (E) HMGB1, and (F) release profiles of 4T1 cells treated with free OXA under hypoxia and PAOL under normoxia and hypoxia. Scale bars were 20 μm. Cell nucleus was stained with DAPI. (G) The maturation DCs rate of BMDC co-incubated with 4T1 cells with different treatments. (H) Flow cytometric examination of DC maturation. Data are presented as mean ± SD (*n* = 3).

The cellular uptake was evaluated by the detection of Pt. As shown in Figure 3(A), PAOL achieved significantly higher cellular uptake of Pt in 4T1 cells compared with free OXA under hypoxia condition, which could be attributed to the efficient endocytosis of the micelles and the passive diffusion of OXA prodrug in contrast to the only passive diffusion of free OXA. PAOL under hypoxia also showed a much higher cellular uptake than under normoxia condition. It could be ascribed to the hypoxia-triggered PEG removal and the released OXA prodrug could also diffused into the tumor cells.

The OXA (IV) prodrug could be reduced to active OXA by endogenous GSH and killed the tumor cells. In consequence, the cytotoxicity was detected by MTT assay against 4T1 cells in both normoxia and hypoxia condition. Both free OXA and PAOL showed concentration-dependent cytotoxicity pattern (Figure 3(B,C)). The IC_50_ of OXA under normoxia and hypoxia condition was quantified to be 9.83 × 10^−6^ M and 15.49 × 10^−6^ M, respectively. Meanwhile, the IC_50_ of PAOL under normoxia and hypoxia condition was quantified to be 6.10 × 10^−6^ M and 3.94 × 10^−6^ M, respectively. The cytotoxicity decreased under hypoxia than normoxia as the concentration of free OXA increased, because the hypoxic cancer cells are less sensitive to OXA than normoxic cancer cells (Cao et al., 2020). To the contrary, PAOL exhibited higher cytotoxicity under the hypoxia condition than that under normoxia condition, suggesting the efficiency of the hypoxia-responsive cleavage of the micelles.

### 3.3. ICD induction *in vitro*

Previous researches have reported that OXA can induce ICD of various tumor cells through releasing DAMPs (Lu et al., 2017; Feng et al., 2018), which is usually characterized by the exposure of CRT, the release of high mobility group box 1 (HMGB1) and adenosine triphosphate (ATP). The exposed CRT on the plasma membrane acts as an ‘eat me’ signal for DCs and mediate immunogenicity of the tumors (Rodriguez-Ruiz et al., 2019). The release of HMGB1 can elicit DC activation and facilitate the antigen presentation of DCs to T cells (Zhou et al., 2019). ATP can trigger the recruitment of the antigen-presenting cells (APCs) to promote the phagocytosis of the dying tumor cells by APCs (Krysko et al. 2012). CLSM was utilized to observed the exposure of CRT and the release of HMGB1. As shown in Figure 3(D,E), PAOL and OXA exhibited significantly exposure of CRT and the release of HMGB1. PAOL exhibited discrepant expression under normoxia and hypoxia condition. Both the endocytosis of PAOL and the passive diffusion of released OXA prodrug under hypoxia condition promoted the cellular of OXA and further enhanced the release of CRT and HMGB1. The release profile of ATP was tested with a commercial ATP assay kit, and both OXA and PAOL could induce ATP secretion of tumor cells (Figure 3(F)). 4T1 cells incubated with PAOL under hypoxia condition showed more extracellular secretion of ATP than under normoxia condition. These results support that PAOL could effectively induce ICD of tumor cells and especially under hypoxia condition.

Furthermore, we also investigated ICD-induced DC maturation to further evaluate the immunogenicity of the tumor cells induced by PAOL. The markers of CD80 and CD86 were used to characterize the maturations of DCs. BMDCs were incubated with tumor cells pretreated with OXA and PAOL under different conditions. Both OXA and PAOL could induce the activation of DCs. Nevertheless, tumor cells treated with PAOL under hypoxia condition could effectively promote the maturation of DCs which is more significant than that with OXA and PAOL under normoxia condition (Figure 3(G,H)). These results suggested that PAOL could induce ICD of tumor cells effectively and the ICD-induction efficacy of PAOL could be improved further under hypoxia condition.

### 3.4. Biodistribution

**Figure 4.**
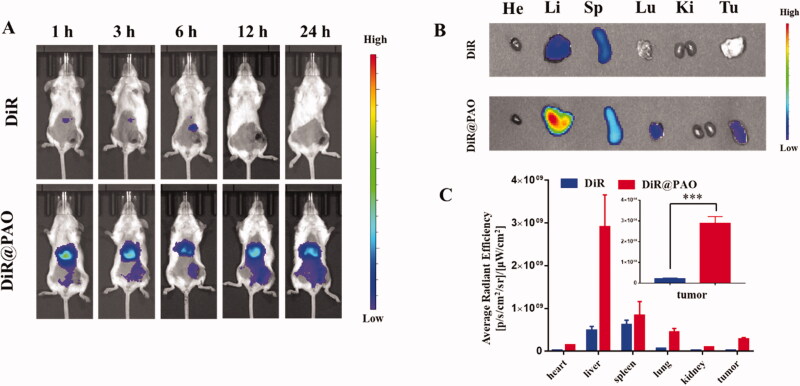
Biodistribution of PAO *in vivo*. (A) Fluorescence imaging of free DiR and DiR labeled PAO distribution in 4T1 tumor-bearing mice *in vivo*. (B) *Ex vivo* fluorescence imaging of the major organs of heart (He), liver (Li), spleen (Sp), lung (Lu), kidney (Ki), and the tumor (Tu) at 24 h postinjection. (C) Quantitative analysis of average radiant efficiency. Data are presented as mean ± SD (*n* = 3).

Near infrared dye DiR was loaded with PAO to evaluate the tumor accumulation by using an IVIS spectrum small-animal imaging system. It is shown in Figure 4(A) that the tumor-bearing mice with intravenous injection of DiR@PAO showed gradually increased fluorescence signals at the tumor site. The mice were sacrificed after 24 h postinjection, and the tumors and main organs (heart, liver, spleen, lung, and kidney) were imaged and quantitated (Figure 4(B,C)). Strong florescence signals were observed in the liver in both DiR and DiR@PAO and it reflects that the free DiR and the micelles are mainly eliminated by macrophage cells in the liver (Zhang et al., 2020). The fluorescence signals of DiR@PAO at the tumor site was 14.58-fold higher than that of free DiR, which highlights the tumor accumulation and retention of PAOL.

### 3.5. Antitumor and antimetastasis effects *in vivo*

**Figure 5.**
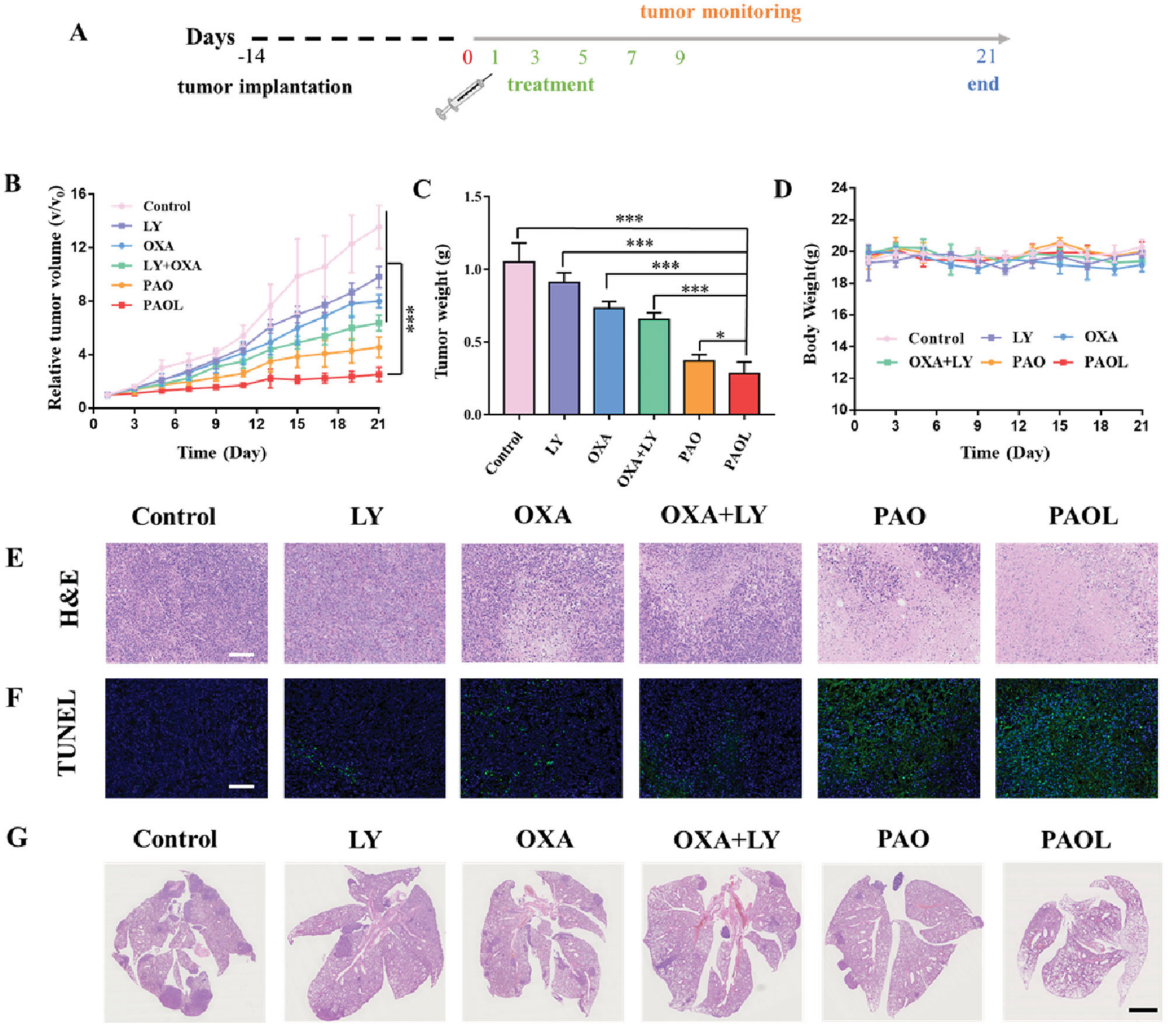
Antitumor effect of PAOL *in vivo*. (A) The therapeutic schedule for the treatments. (B) The tumor growth cures in 4T1 tumor-bearing mice following the indicated treatments. (C) The tumor weight after the treatments. (D) The body weight of the mice during the treatments. (E) H&E staining and (F) TUNEL assay of the tumors after the different treatments. Scale bars were 100 μm. (G) H&E staining of the lungs after different treatments. Scale bars were 2.5 mm. Data are presented as mean ± SD (*n* = 6).

The antitumor effect was evaluated in 4T1 tumor-bearing mice (Figure 5(A)). A total of 36 tumor-bearing mice were randomly divided into six groups and were treated with different administrations. The weight of the mice and the volume of the tumors were recorded every two days. As shown in Figure 5(B), combination with OXA and LY showed increased tumor regression efficacy compared with monotherapy, which displayed moderate antitumor effect. In addition, PAOL showed the best treatment effect, which may be caused by the effective tumor accumulation and the effect of combination therapy. At the end of the treatments, the mice were sacrificed and the weight of the tumors were measured. The average weight of the tumors treated with PAOL was obviously reduced compared to that of other groups (Figure 5(C)). Meanwhile, no significant weight loss was observed during the treatments, indicating a negligible side effect of PAOL for the treatment. (Figure 5(D)).

To further determine the therapeutic efficacy of PAOL, H&E and TUNEL staining assays were used to detect the histological images and cell apoptosis in tumor tissues (Figure 5(E,F)). The control group showed the morphology of high proliferative tumor cells with large and deeply stained nucleus. On the contrary, the PAOL treatment group displayed shrunk nuclei and the largest necrosis area. Besides, the TUNEL assay showed the largest number of apoptotic cells in PAOL treatment group, which is consistent with the result of H&E staining. Meanwhile, other treatment groups induced moderate tumor apoptosis and necrosis compared with the control group.

**Figure 6.**
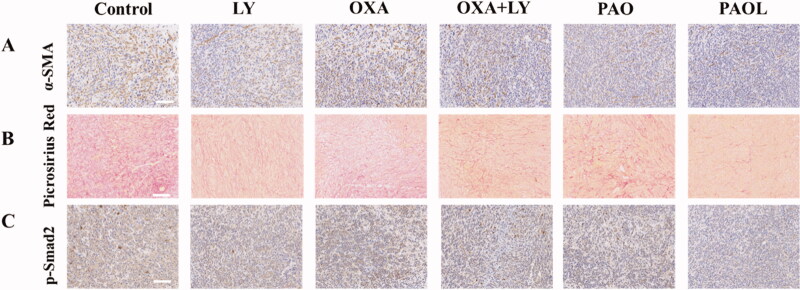
(A) α-SMA immunohistochemistry and (B) Picrosirius Red staining of the tumor slices. (C) The p-Smad2 immunohistochemical staining of the tumors after the different treatment.

Metastasis is a major cause of mortality in cancer patients (Zhang et al., 2020). TGF-β is related not only with tumor progression, but also with epithelial–mesenchymal transitions (EMTs) and tumor metastasis. Therefore, the antimetastasis effect of PAOL was investigated *in vivo* as well. As lung is a common metastatic site for 4T1 tumor model, metastatic nodules of 4T1 tumor cells in the lung were observed by H&E staining. The PAOL treated group could significantly inhibit the formation of metastatic nodules in the lung while other groups demonstrated different degrees of metastatic nodules (Figure 5(G)). Due to the important role of cancer-associated fibroblasts (CAFs) (Lan et al., 2018) and ECM (Gilkes et al., 2014) in promoting metastasis, we further performed α-SMA immunohistochemistry assay and Picrosirius Red staining to illustrate the anti-metastasis mechanism of PAOL. α-SMA is a marker of CAFs activation and Picrosirius Red staining was used to evaluate the deposition of collagen in tumors. As shown in Figure 6(A,B), PAOL could effectively reduce α-SMA expression and collagen deposition relative to control and other treatment groups as expected. The TGF-β receptor kinase inhibitor LY could reduce p-Smad2 expression (Flechsig et al., 2012); therefore, the immunohistochemistry of p-Smad2 was also performed. As shown in Figure 6(C), the expression of p-Smad2 in PAOL group was decreased significantly compared with other groups. Overall, blockade of TGF-β signaling could reduce tumor stroma content, expression of ECM proteins, and fibroblast activation (Lan et al., 2018).

Moreover, OXA is a platinum-based chemotherapeutic agent which is used for the treatment of colorectal tumor model (Feng et al., 2018; Shen et al., 2020). We also evaluated the antitumor effect of PAOL against murine CT26 colorectal cancer and the antitumor efficiency is consistent with 4T1 tumor model. The PAOL treatment group showed the highest antitumor efficacy without causing obvious body weight loss (Figures S14, S15) and concordant results were demonstrated by H&E staining of the CT26 tumors (Figure S16).

### 3.6. *In vivo* antitumor immune response

**Figure 7.**
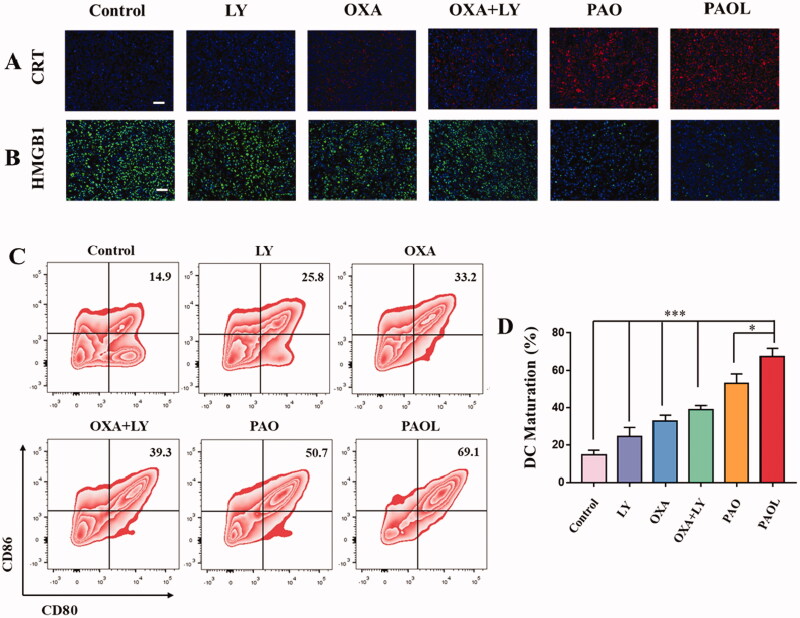
ICD effect *in vivo*. (A) CRT expression and (B) HMGB1 release profiles on the tumor slices collected from the 4T1 tumor-bearing mice with different treatments as indicated. (C) The flow cytometric analysis of DC maturation in lymph nodes with various treatments. The quantitative flow cytometric analysis of (D) maturation DCs (CD11^+^CD80^+^CD86^+^). Data are presented as mean ± SD (*n* = 3).

The significant therapeutic performance of PAOL might be closely associated with the antitumor immune response, which was induced by ICD and blockade of TGF-β signaling. We therefore evaluated the immune mechanism behind the excellent synergistic antitumor efficacy. *In vitro* experiments showed that PAOL could induce robust ICD effect. In consequence, we first examined the immunofluorescence of CRT and HMGB1 in the tumor sections. The 4T1 tumor-bearing mice were given the same treatments as described in the antitumor study. After that, the tumor tissues were harvested for examination of the CRT expression and HMGB1 release. As shown in Figure 7(A,B), predominant CRT expression and HMGB1 release were observed in PAO and PAOL group, both of which were much higher than that in the other groups. The results were indicative of effective ICD of cancer cells after PAOL treatment *in vivo*. The released DAMPs and antigens would recruit DCs to the tumor site and activate systemic immune response. Therefore, the draining lymph nodes were harvested simultaneously to analyze the DC maturation by flow cytometry. It is shown in Figure 7(C,D) that the maturation of DCs post PAOL injection was 69.1%, which is 2.1- and 2.68-fold higher than that in mice treated with free OXA and LY, respectively.

**Figure 8.**
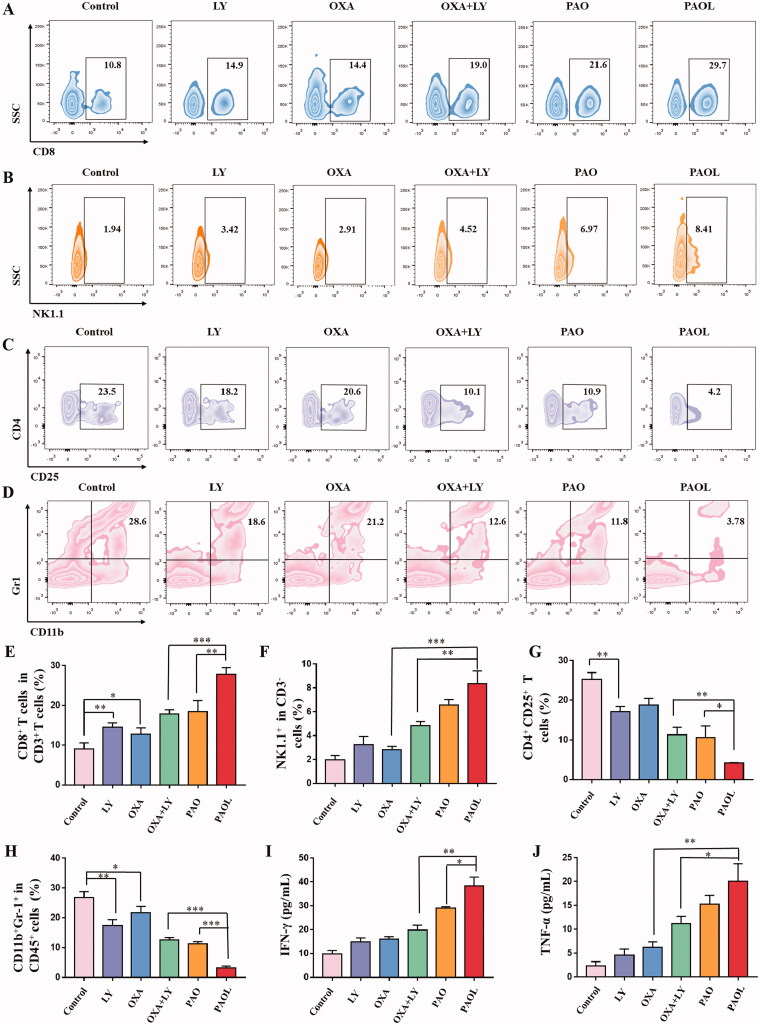
Antitumor immune mechanism of the treatments. Flow cytometric analysis of the frequencies of (A) CD8^+^ T cells in CD3^+^ T cells, (B) NK cells (NK1.1^+^ in CD3^–^ cells), (C) Tregs (CD4^+^ CD25^+^ T cells), (D) MDSCs (CD11b^+^Gr1^+^ in CD45^+^ cells). The quantitative flow cytometric analysis of (E) CD8^+^ T cells in CD3^+^ T cells, (F) NK cells, (G) Tregs, and (H) MDSCs. The secretion level in the serum of (I) IFN-γ and (J) TNF-α. Data are presented as mean ± SD (*n* = 3).

Subsequently, we also analyzed the proportion of tumor intratumoral infiltration of tumor-infiltrating CD8^+^ cytotoxic T cells (CTLs), NK cells, immunosuppressive Tregs and MDSCs after the administration of different treatments. TGF-β represses CD8^+^ T cell-mediated antitumor immunity (Derynck et al., 2021). Flow cytometry analysis of tumor cells showed that PAOL treatment could significantly recruit CD8^+^ T cell (Figure 8(A,E)) and NK cells ((Figure 8(B,F)) compared with the other groups. The immunosuppressive cells were also detected, the results demonstrated that PAOL could effectively reduce the number of Tregs (Figure 8(C,G)) and MDSCs (Figure 8(D,H)) in tumor microenvironment. Macrophages possess M1 and M2 phenotypes. M1 macrophage exhibits pro-inflammatory activity and M2 macrophage exhibits anti-inflammatory activity (Zhu et al., 2020). Meanwhile, as iNOS and Arginase1 (Arg1) are the biomarkers of M1- and M2-like tumor associated macrophages (Wang et al., 2020), we also examined the ratio of M1/M2-TAMs. The similar results were obtained as other immune cells, the PAOL treatment group could improve the ratio of M1/M2 more effectively than other groups (Figures S17, S18).

The secretion of cytokine plays an important role in T cell-mediated antitumor immunity (Lan et al., 2020), such as IFN-γ and TNF-α. As shown in Figure 8(I,J), PAOL treatment group could obviously boost the secretion of IFN-γ and TNF-α. The levels of the cytokines were much higher than other groups and were consistent with the tumor rejection. The results further validated PAOL could effectively trigger antitumor immune response.

Overall, all the above results demonstrated that PAOL could be accumulated into tumors efficiently through EPR effect. Subsequently, the micelles cleaved at the hypoxia tumor site and LY was released. The released LY could remodel the tumor environment by inhibition of TGF-β mediated immunosuppression. Meanwhile, the OXA prodrug was reduced to OXA and could directly kill tumor cells and elicit antitumor response by ICD effect. The synergistic therapeutic strategy could restrain tumor growth and metastasis effectively to improve chemoimmunotherapy.

### 3.7. *In vivo* safety evaluation

To evaluate the potential *in vivo* systematic toxicity of the different treatments, serum chemistry and H&E section of major tissues were studied. No morphological difference in major organs (heart, liver, spleen, lung, and kidney) was observed (Figure S19). Besides, there was no significant difference of the organ coefficient between the treated groups and the control group (Figure S20). Alanine aminotransferase (ALT) and aspartate aminotransferase (AST) were tested to observe the hepatic toxicity. Meanwhile, creatinine (CREA) and urea nitrogen (UREA) were tested to observe the kidney toxicity. As shown in Figure S21, there was no obvious difference between the treated groups and the control group. These results indicated that PAOL presents high biosafety and possesses potential application in cancer therapy.

## 4. Conclusions

In summary, a tumor hypoxia and reduction dual activatable nanosystem was developed for enhanced chemoimmunotherapy by triggering the ICD effect of tumor cells and remolding the tumor environment. Our results verified that the prepared PAOL could passively accumulate at the tumor site and effectively release LY and OXA. As a result, it exhibited significant therapeutic effect toward mouse models of breast and colorectal cancer by eliciting effective antitumor immunity by ICD effect and TGF-β blockade immune suppression. This work provided a promising strategy with the cooperation of chemotherapy and TGF-β receptor kinase inhibitor for efficient cancer chemoimmunotherapy.

## Supplementary Material

Supplemental Material
