# Supplementary material for: Dual-responsive nanosystem based on TGF-β blockade and immunogenic chemotherapy for effective chemoimmunotherapy
Source: Drug Deliv. 2022 May 4;29(1):1358–69. doi: 10.1080/10717544.2022.2069877 (PMC9090387; doi:10.1080/10717544.2022.2069877)
Supplement: Supplemental Material [file IDRD_A_2069877_SM5319.docx]

**Supporting Information**

**Synthesis of OXA (IV)-NH_2_**

The synthesis of OXA (IV)-NH_2_ was divided into five steps.

Firstly, oxaliplatin (1g, 2.52 mmol) was suspended in 10 mL of 30% H_2_O_2_ aqueous solution at dark and stirred at room temperature for 24 h. The reaction solution was freeze-dried to obtain compound **1** as yellowish powder (1.085 g, yield 100%). The chemical structure was characterized by ^1^H-NMR and ESI-MS. ^1^H NMR (300 MHz, D2O): 2.84-2.77 (2H, m, H-1,10), 2.30-2.20 (2H, m, H-2,8), 1.64-1.48 (4H, m, H-4~7), 1.28-1.16 (2H, m, H-3,9).

Then the reaction was followed by the reported steps. Compound **1** (0.5 g，1.16 mmol)and succinic anhydride (0.116 g，116 mmol) were dissolved in 10 mL of anhydrous dimethyl sulfoxide (DMSO) at dark and stirred at room temperature. After 24 h, the solution was lyophilized and redissolved in methanol and precipitated in cold ethyl ether to obtain compound **2** as white powder (0.554 g, yield 90%).^1^H NMR (300 MHz, DMSO), 2.88~2.76 (m, 2H, H-1,10), 2.66~2.52 (m, 4H, H-11,12), 2.33~2.18 (m, 2H, H-2,8), 1.67~1.45 (m, 4H, H-4~7), 1.26~1.16 (m, 2H, H-3,9).

Stearic anhydride (975 mg,1.77 mmol) was added into the solution of compound **2**(781 mg,1.47 mmol) in 10 mL of DMF. The solution was stirred under dark at 60 ℃ for 24 h. After cooling to room temperature, the mixture was condensed by vacuum evaporation and purified by column chromatography to obtain compound **3** (504.0 mg, yield 43%). ^1^H NMR (300 MHz, DMSO), 0.88~0.81 (t, 3H, H-47~49), 1.19~1.05 (m, 4H, H-4~7), 1.31~1.13 (m, 28H, H-19~46), 1.45~1.31 (m, 4H, H-2,3,8,9), 1.54~1.45 (m, 2H, H-17,18), 2.17~2.04 (m, 2H, H-1,10), 2.28~2.17 (m, 2H, H-13,14), 2.43~2.29 (m, 2H, H-11,12), 2.65~2.51 (m, 2H, H-15,16). ESI-MS [M+H]^+^: Calc.798.34, found 798.35.

Afterwards, EDCI (146.1 mg, 0.762 mmol), HOBT (102.9 mg, 0.762 mmol) and N-BOC-ethylenediamine were added to a solution of compound **3** (405 mg, 0.507 mmol) in 10 mL of DMF. The mixture was stirred under dark at 70 ℃ overnight. After cooling to room temperature, the mixture was condensed by vacuum evaporation and purified by column chromatography to obtain compound **4** (371.5 mg, yield 78%). ^1^H NMR (300 MHz, DMSO), 0.90~0.78 (t, 3H, H-62~64), 1.18~1.02 (t, 2H, H-4,6), 1.30~1.15 (t, 28H, H-34~61), 1.35~1.30 (t, 2H, H-5,7), 1.39~1.34 (s, 9H, H-21~29), 1.59~1.37 (m, 6H, H-2,3,8,9,32,33), 2.21~2.0 (m, 2H, H-1,10), 2.32~2.18 (m, 4H, H-11~14), 2.61~2.51 (m, 2H, H-30,31), 3.06~2.90(m, 4H, H-16~19), 6.91~6.71 (m, 1H, H-20), 7.96~7.76 (m, 1H, H-15). ESI-MS [M+H]^+^: Calc. 940.45, found 940.46.

Finally, compound **4** (200 mg, 0.213mmol) was dissolved in 2 mL of dichloromethane and 6 mL of TFA was added. The mixture was reacted at room temperature for 4 h. The mixture was condensed by vacuum evaporation and purified by column chromatography to obtain compound **5** (169.8 mg,95%). ^1^H NMR (300 MHz, DMSO), 0.90~0.80 (t, 3H, H-54~56), 1.19~1.09 (m, 2H, H-4,6), 1.29~1.16 (m, 28H, H-26~53), 1.37~1.27 (m, 2H, H-5,7), 1.57~1.37 (m, 6H, H-2,3,8,9,24,

25), 2.20~2.03 (m, 2H, H-1,10), 2.36~2.18 (m, 4H, H-11~14), 2.65~2.54 (m, 2H, H-22,23), 2.89~2.81 (m, 2H, H-20,21), 3.30~3.20 (m, 4H, H-16~19), 8.07~8.03 (m, 1H, H-15). ESI-MS [M+H]^+^: Calc. 840.40,found 840.41.

**Synthesis of PEG-AZO-COOH**

The reaction was reacted by previous method^5^. Azobenzene-4,4′-dicarboxylic acid (102 mg,0.375 mmol), EDCI (47.93 mg,0.25 mmol), NHS (28.77 mg,0.25 mmol) were dissolved in 25 mL of pyridine and PEG_2k_-NH_2_ (500 mg,0.25mmol) was added. The reaction was stirred overnight at room temperature. The mixture was condensed by vacuum evaporation and purified to obtain PEG_2k_-AZO-COOH (306.2 mg, yield: 54%). ^1^H NMR (300 MHz, CDCl_3_):3.37~3.35 (s), 3.64~3.61 (m), 8.03~7.91 (m),

8.22~8.15 (m).

**Synthesis of PEG-AZO-OXA**

Briefly, PEG-AZO-COOH (290 mg,0.13 mmol), EDCI (37 mg,0.19 mmol)、NHS(22.20 mg, 0.19 mmol) were dissolved in 10 mL of DMSO at room temperature and then compound **5** (129.70 mg,0.16 mmol)was added and the mixture was stirred at dark. After reacting for 24 h, the solution was dialyzed (MW 1000) for 48 h. The final product was lyophilized to obtain PEG_2k_-AZO-OXA (326.25mg, yield 87%). 0.87~0.82 (t, 3H), 1.14~1.05 (m, 2H), 1.29~1.12 (m, 28H), 1.34~1.24 (m, 2H), 1.57~1.29 (m, 6H), 2.11~1.94 (m, 2H), 2.34~2.15 (m, 4H), 2.60~2.51 (m, 2H), 2.87~2.58 (m, 4H), 3.24~3.22 (s, 3H), 3.60~3.32 (m, 180H), 8.13~7.89 (m, 7H), 8.76~8.70 (m,1H).

**Figure S1.** The synthesis routes of OXA(Ⅳ)-NH_2._

**Figure S2.** The synthesis routes of PEG-AZO-COOH.

**Figure S3.** The synthesis routes of PEG-AZO-OXA.


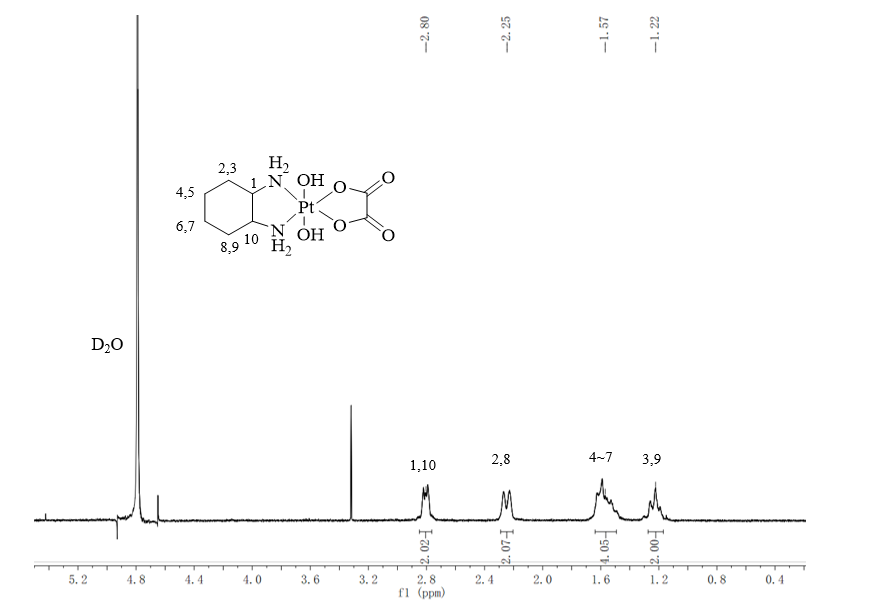


**Figure S4.** The ^1^H-NMR spectrum of Compound 1.


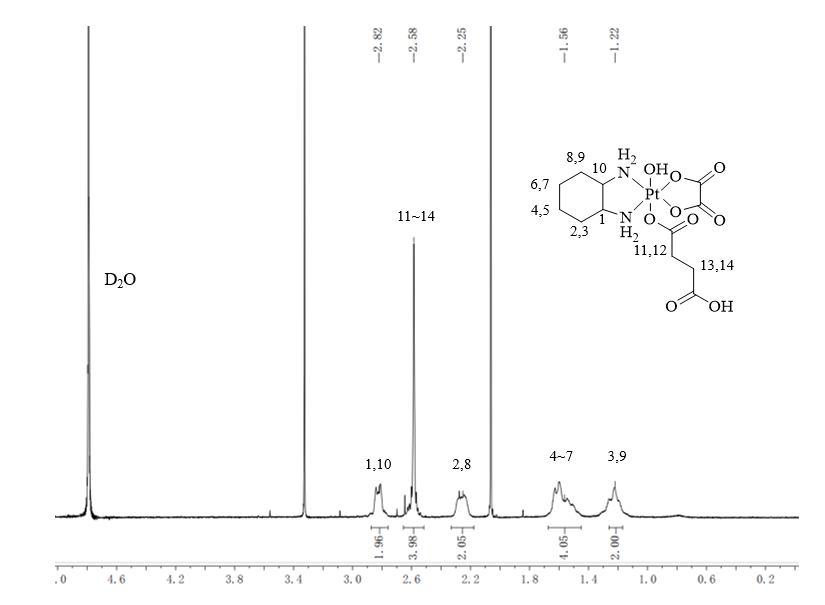


**Figure S5.** The ^1^H-NMR spectrum of Compound 2.


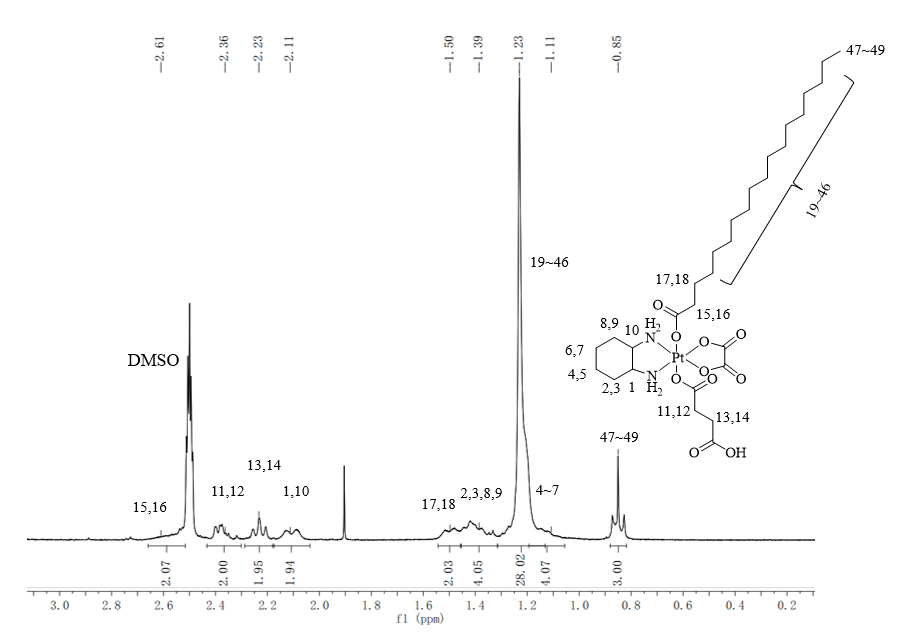


**Figure S6.** The ^1^H-NMR spectrum of Compound 3.


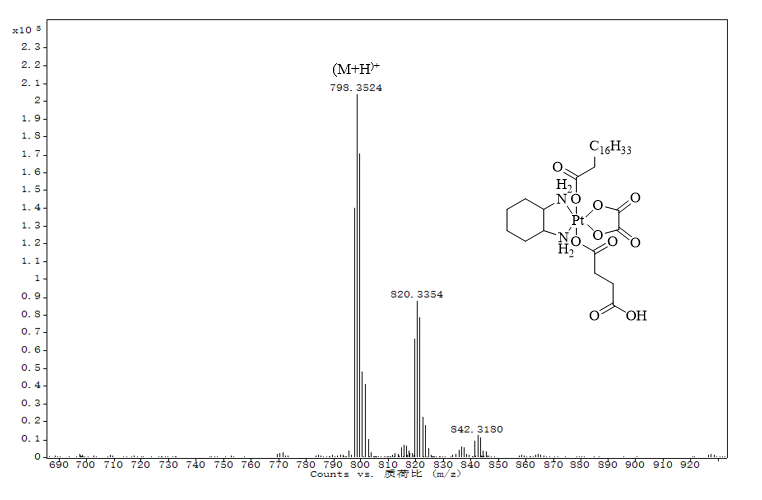


**Figure S7.** The ESI-MS spectrum of Compound 3.


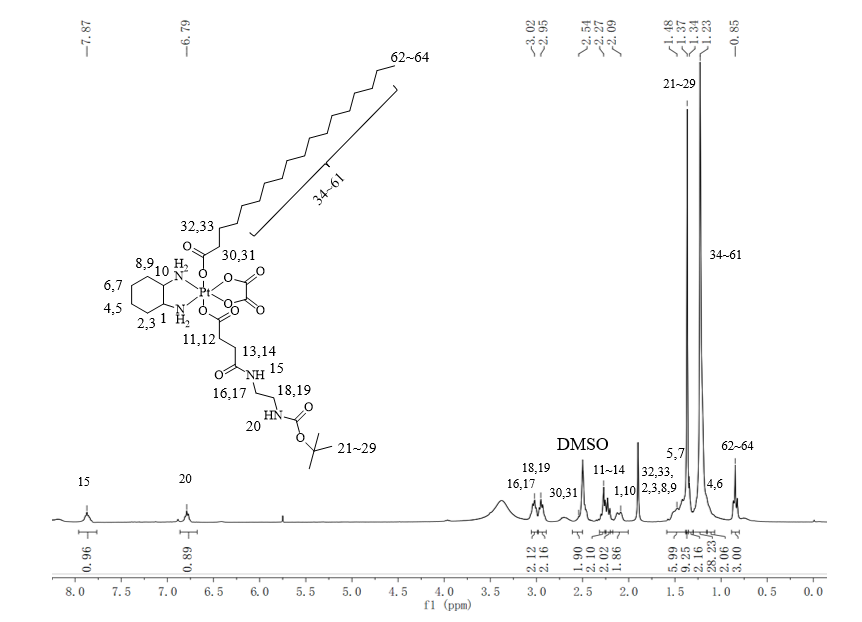


**Figure S8.** The ^1^H-NMR spectrum of Compound 4.


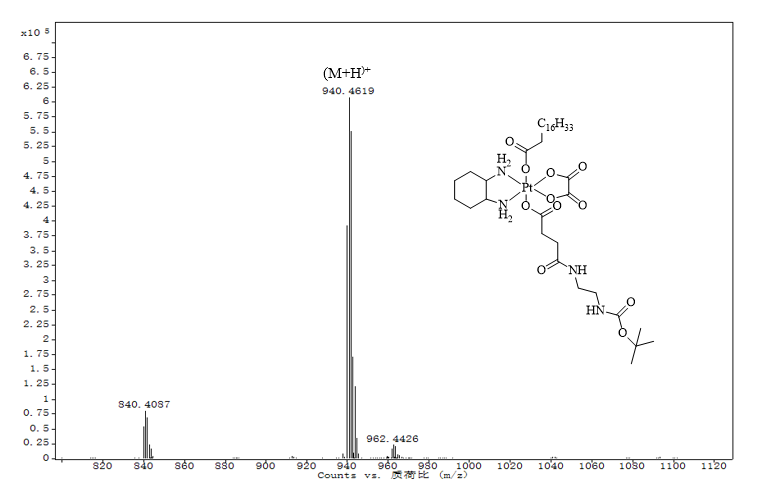


**Figure S9.** The ESI-MS spectrum of Compound 4.


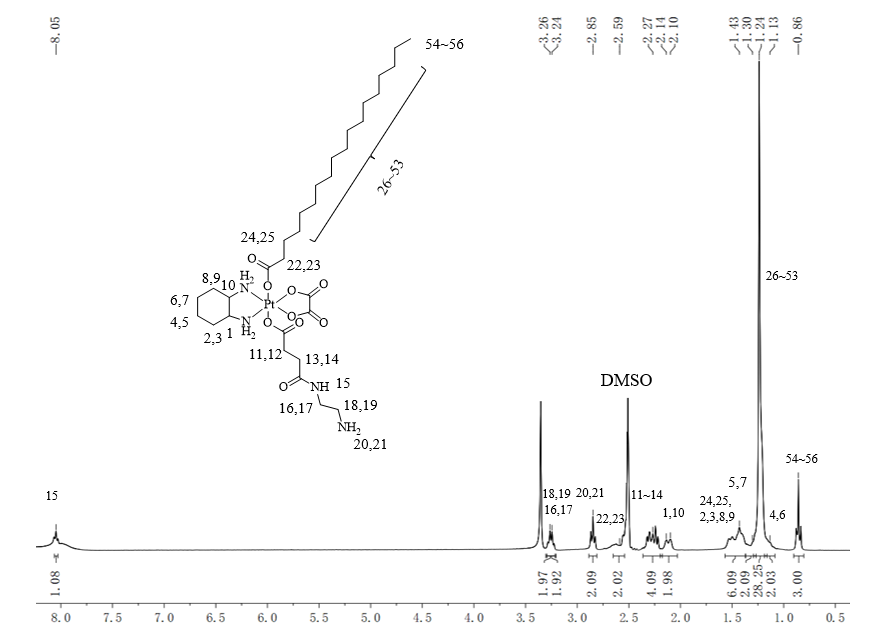


**Figure S10.** The ^1^H-NMR spectrum of Compound 5.


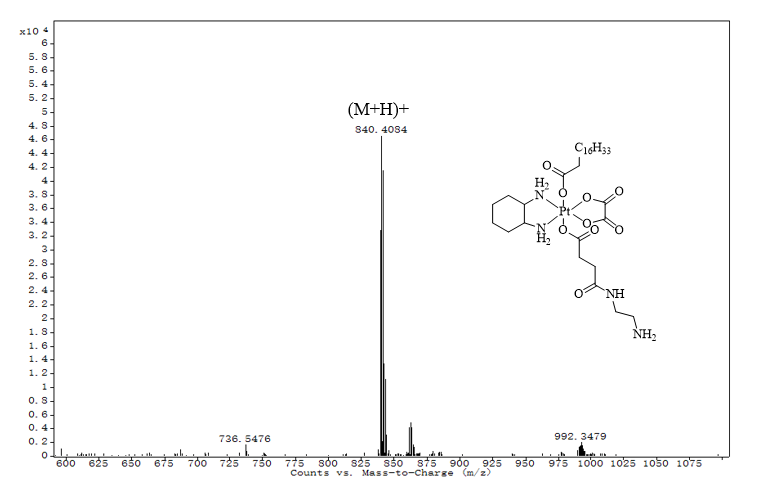


**Figure S11.** The ESI-MS spectrum of Compound 5.


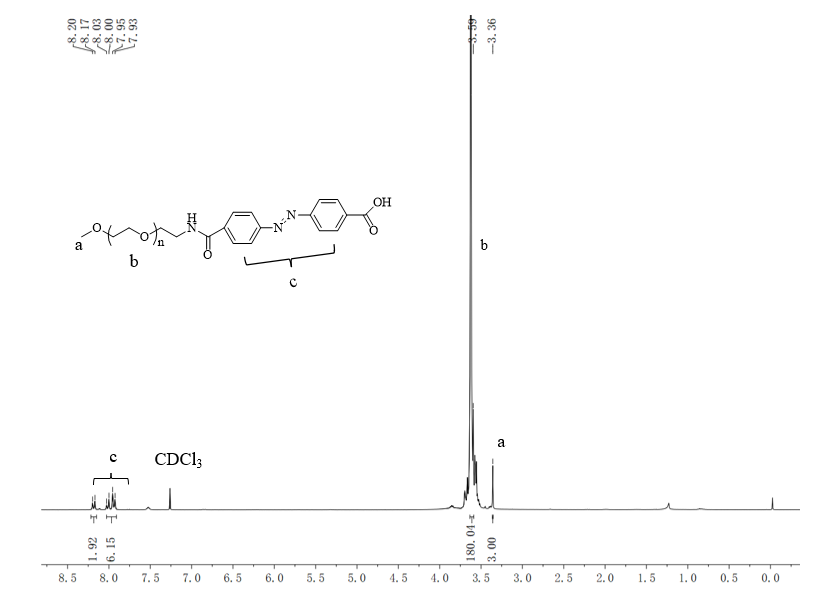


**Figure S12.** The ^1^H-NMR spectrum of PEG-AZO-COOH.


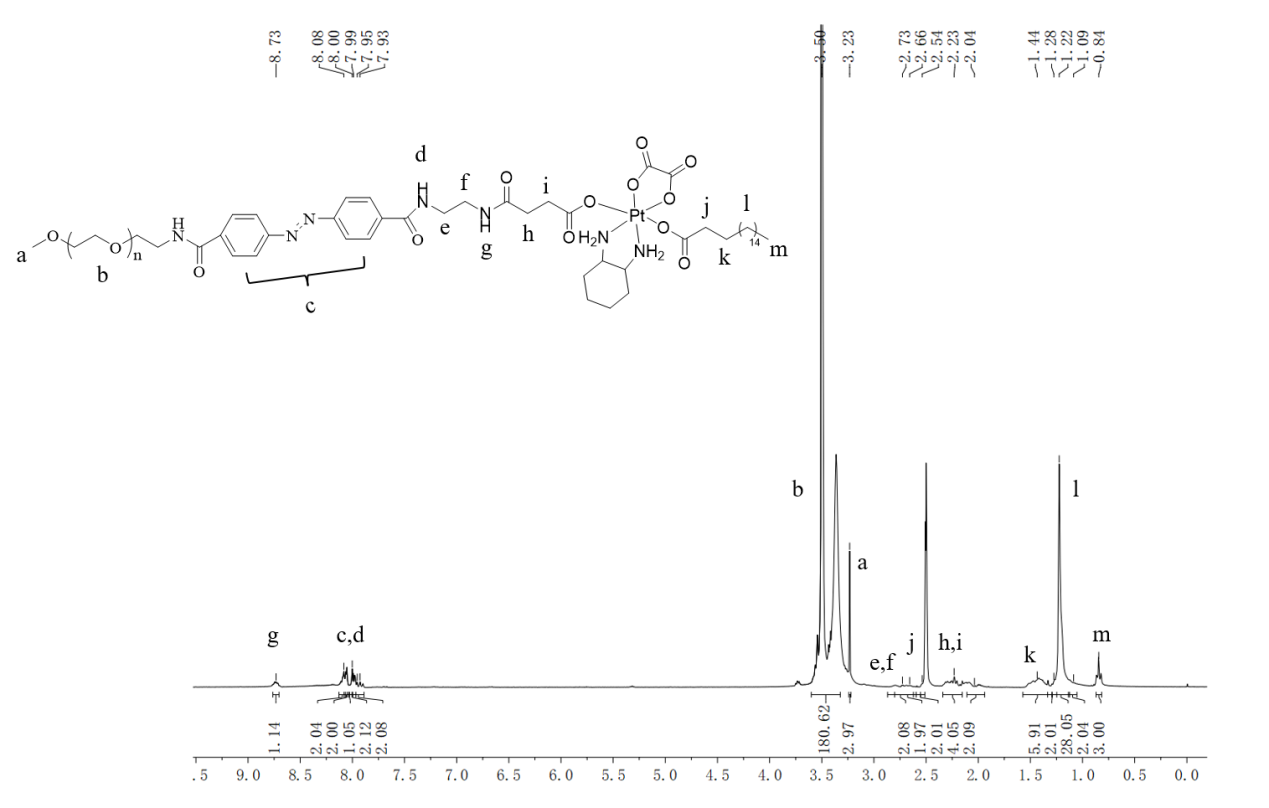


**Figure S13.** The ^1^H-NMR spectrum of PEG-AZO-OXA.

**
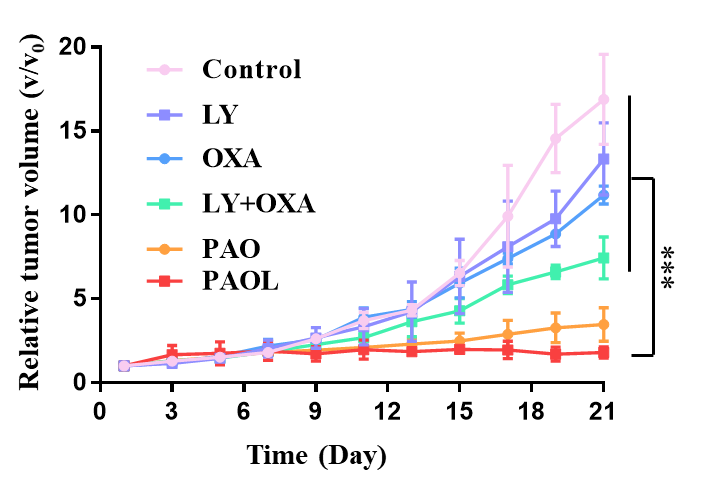
**

**Figure S14.** The tumor growth curves of CT26 tumor-bearing mice.


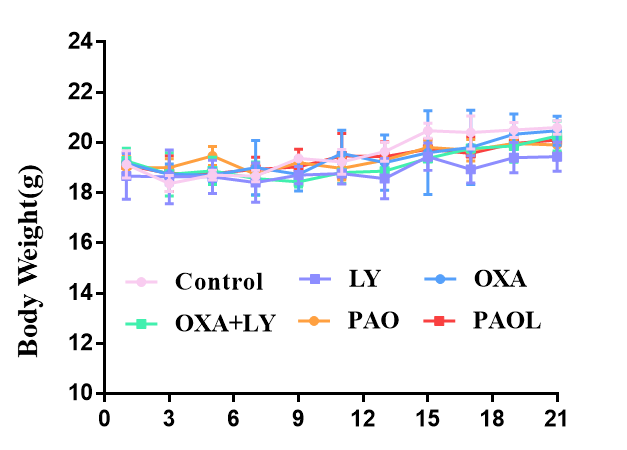


**Figure S15.** Body weight of the CT26 tumor-bearing mice.


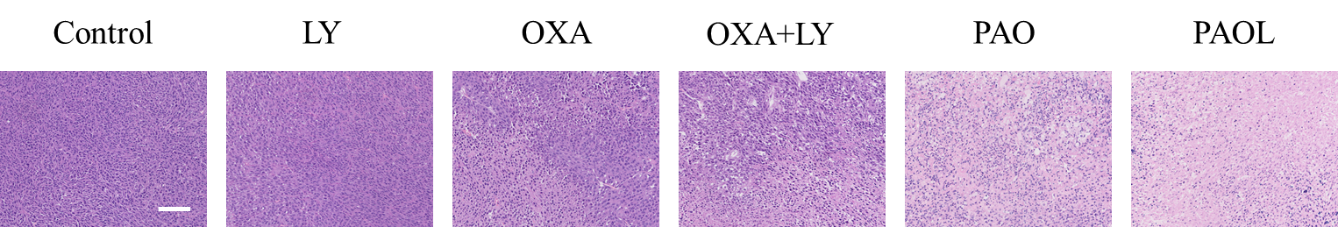


**Figure S16.** H&E staining of the CT26 tumors after different treatments.


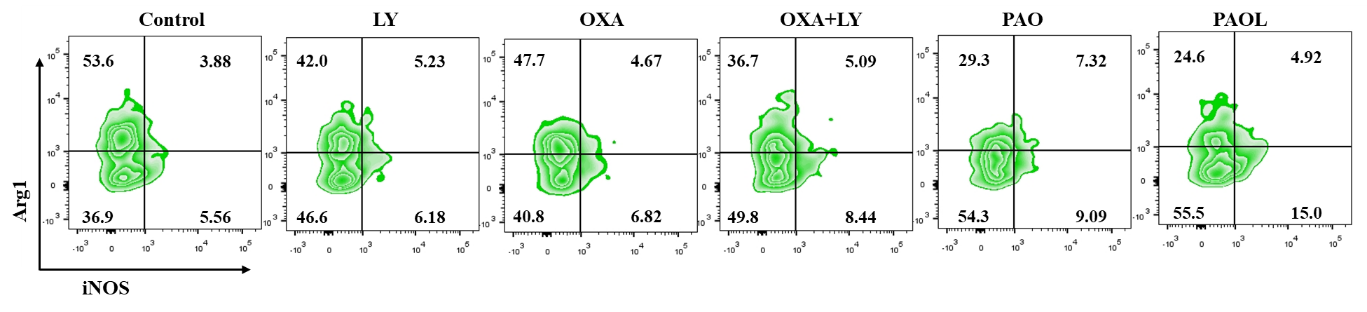


**Figure S17.** The proportions of TAM cells (in CD11b^+^ F4/80^+^cells).


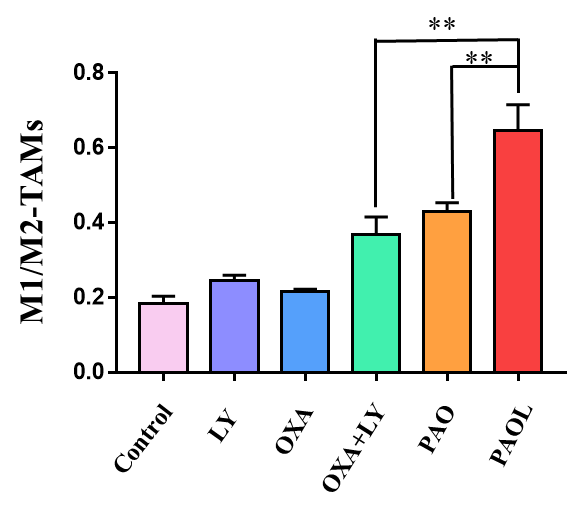


**Figure S18.** The quantitative flow cytometric analysis of TAM cells (in CD11b^+^ F4/80^+^cells).


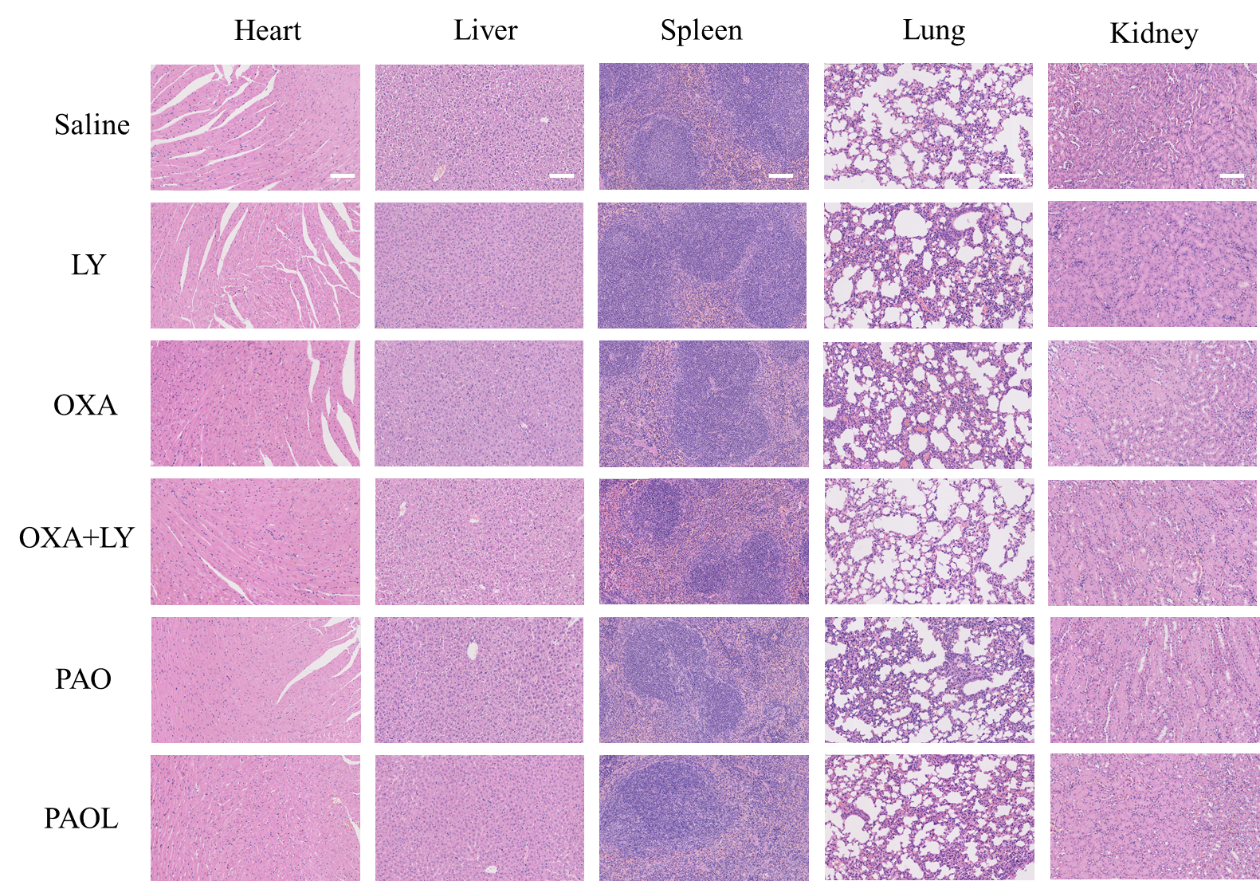


**Figure S19.** H&E morphology of major organs.

_
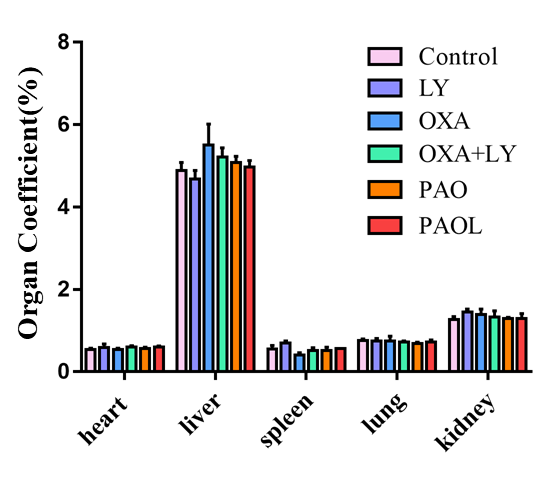
_**Figure S20.** Organ coefficient after various treatments.


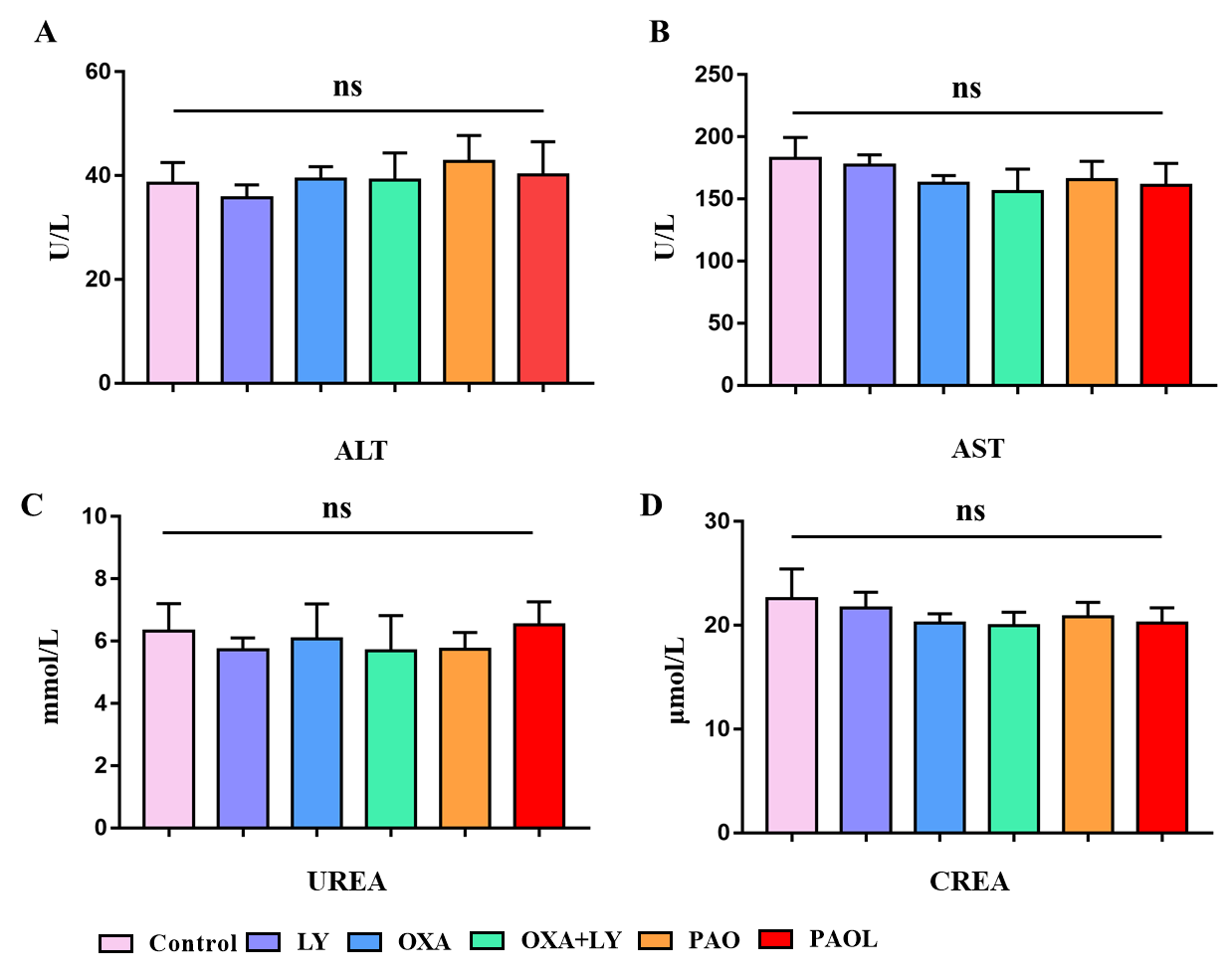


**Figure S21.** Blood biochemical analysis.
